# Large language models for the mental health community: framework for translating code to care

**DOI:** 10.1016/S2589-7500(24)00255-3

**Published:** 2025-01-07

**Authors:** Matteo Malgaroli, Katharina Schultebraucks, Keris Jan Myrick, Alexandre Andrade Loch, Laura Ospina-Pinillos, Tanzeem Choudhury, Roman Kotov, Munmun De Choudhury, John Torous

**Affiliations:** Department of Psychiatry, New York University School of Medicine, New York, NY, USA; Department of Psychiatry, New York University School of Medicine, New York, NY, USA; Partnerships and Innovation, Inseparable, Los Angeles, CA, USA; Laboratorio de Neurociencias (LIM 27), Instituto de Psiquiatria, Hospital das Clinicas HCFMUSP, Faculdade de Medicina, Universidade de Sao Paulo, Sao Paulo, Brazil; Department of Psychiatry and Mental Health, Faculty of Medicine, Pontificia Universidad Javeriana, Bogota, Colombia; Department of Information Science, Jacobs Technion–Cornell Institute, Cornell Tech, New York, NY, USA; Department of Psychiatry, Stony Brooks University, Stony Brooks, NY, USA; School of Interactive Computing, College of Computing, Georgia Institute of Technology, Atlanta, GA, USA; Department of Psychiatry, Beth Israel Deaconess Medical Center, Harvard Medical School, Boston, MA, USA

## Abstract

Large language models (LLMs) offer promising applications in mental health care to address gaps in treatment and research. By leveraging clinical notes and transcripts as data, LLMs could improve diagnostics, monitoring, prevention, and treatment of mental health conditions. However, several challenges persist, including technical costs, literacy gaps, risk of biases, and inequalities in data representation. In this Viewpoint, we propose a sociocultural–technical approach to address these challenges. We highlight five key areas for development: (1) building a global clinical repository to support LLMs training and testing, (2) designing ethical usage settings, (3) refining diagnostic categories, (4) integrating cultural considerations during development and deployment, and (5) promoting digital inclusivity to ensure equitable access. We emphasise the need for developing representative datasets, interpretable clinical decision support systems, and new roles such as digital navigators. Only through collaborative efforts across all stakeholders, unified by a sociocultural–technical framework, can we clinically deploy LLMs while ensuring equitable access and mitigating risks.

## Introduction

WHO has highlighted the urgent need for transformation in mental health care globally. Conditions such as depression, anxiety, and psychosis are the leading causes of disability, affecting 970 million people worldwide with an economic impact of US$1 trillion annually.^1^ This crisis is deepened by considerable gaps in the detection and treatment of mental health conditions. WHO emphasises how digital technologies could help to meet mental health needs across populations at scale,^2^ particularly in underserved areas where the availability of mobile technology surpasses that of traditional health care.^1^ The accelerating enthusiasm for artificial intelligence and large language models (LLMs), exemplified by the widespread adoption of ChatGPT (OpenAI, San Francisco, CA, USA) by the public, has broadened interest in their mental health applications.^3^ To translate this enthusiasm into tangible innovations, first understanding the actual capabilities of LLMs and then identifying how they can be realistically deployed for research and care is crucial. In this Viewpoint, we adopt a sociocultural–technical lens to highlight opportunities and key challenges of the use of LLMs for mental health and offer recommendations for a framework that can help towards realising these opportunities.

## Potential of LLMs for mental health

Mental health conditions are often both identified and treated through language, making them an ideal target for LLMs. Efforts in prevention, early diagnosis, monitoring, and even treatment are all potential areas in which LLMs can augment care and research. However, the mental health implications of LLMs can only be understood in the context of what LLMs can and cannot do. LLMs process unstructured text for both input and output and can be further adapted for medical domains.^4^ LLMs require extensive training datasets and are best conceived as sophisticated pattern-matching programs: just as autocomplete has been able to predict the next word in text messages for years, LLMs might now be able to write or summarise a clinical note, or predict outcomes based on the content of these notes.^5^ This broader applicability emerges from the pattern-matching core of LLMs, which require billions of examples and parameters on which to train. Research findings from peer-reviewed and preprint studies suggest that LLMs could assist in clinical tasks, including diagnostic assessments,^6^ intervention delivery,^3^ and empathic support.^7^

## Problems

Despite their potential, the clinical deployment of LLMs is hindered by several challenges. Firstly, transparency issues arise from the datasets on which these models are trained, hindering multilingual performance^8^ and potentially embedding hidden biases.^3^ Mitigation would require regular monitoring of where data are being sourced from, the data type, how to track the diversity of data sources, and the consideration of inclusion strategies. Furthermore, the technical cost associated with LLMs poses considerable accessibility and implementation barriers, particularly in low-resource settings. These barriers include increasing the need for computing power, dedicated hardware, and growing energy consumptions and carbon footprints,^9^ all of which risk exacerbating existing global inequalities. Lastly, there is a need for improving literacy on LLMs because the complexity of these models can result in a poor understanding of their operation, and of the strategies required to mitigate misleading outputs. Addressing these challenges is crucial to ensure that LLMs can help to serve mental health needs with effectiveness, fairness, and equity.

## Related work

An expanding interest in LLMs has spurred numerous framework proposals, both published and in preprint.^10–19^ However, during our literature search we identified only one publication directed at mental health^18^ that was focused on ethical domains. Although ethics is also a core component of our proposed sociocultural–technical framework, creating a global repository of health information on which to train a novel LLM to generate new insights, and ensuring a foundation in digital literacy to ensure inclusivity, presents a different approach. But many approaches are necessary and a growing number of initiatives are working to assess the sociotechnical limitations of LLMs in high-risk settings, such as law and medicine, through the design of benchmarks coproduced by technical and domain experts (eg, AdSolve). Although these initiatives have helped to advance our understanding of LLMs in medical settings, there is still a need for a comprehensive framework, such as that proposed in this Viewpoint, which integrates contributions from clinicians, medical and computer scientists, and individuals with lived experiences of mental health, given that such a framework does not exist at this time.^19^ Our work addresses these gaps by proposing a sociocultural–technical framework that involves all these stakeholders to identify key opportunities in deploying LLMs for mental health care and research.

## Key opportunities

The Wellcome Trust and Google partnered to host a convening of a diverse set of clinical researchers, computer scientists, funding agencies, and lived experience experts to identify key points for advancing research utility and clinical deployment of LLMs. The attendees’ recommendations focus on model-agnostic features, given the rapid evolution of LLM architectures that frequently challenge established notions of model capabilities and optimal learning strategies (figure).

### Building a global clinical repository

Building safe and useful LLMs will require a global and multimodal biobank of psychiatric texts (eg, journal articles, text books, and websites), research, patient notes, clinical measures, personal outcome metrics, biomarker data, behavioural signatures, and clinical corpora. Efforts at this foundational level are crucial to design models that can engender trust, reduce bias, and minimise risk while reducing downstream harms. Assuming that an independent non-profit governance structure is established that defines and implements appropriate protections and safeguards against data misuse, this repository could also serve as a transparent resource for training, testing, and benchmarking LLMs for mental health. Specifically, given the sensitive nature of mental health data, the biobank should prioritise strong policies on data usage and protection, a federated database system, and robust cybersecurity protocols. Applying a sociocultural–technical lens, this biobank should be supported with concomitant educational activities to help researchers and clinicians identify cases of optimal use for these data and training in its clinical role. Having shared training and educational resources will also support the implementation of LLMs in low-resource settings with limited technical expertise.

### Designing ecosystems to encourage the ethical usage of LLMs

LLMs are tools that will be able to affect clinical care only if placed in the hands of stakeholders that can optimally and responsibly use them. Although past efforts at learning health-care systems in mental health have not transformed the field,^20^ LLMs present an ideal opportunity to create more impactful systems in which the technology and cases of clinical use develop synergistically. LLMs can be used as tools to help facilitate communication and shared decision making between patients and clinicians. For example, LLMs are already being used to help make clinical notes more accessible to patients, and patients could use LLMs to help them practise therapy skills, challenge negative assumptions, or even for reality testing in which users can assess the relative objectivity of some thoughts. These applications and their model training should safeguard sensitive patient information by anonymisation and adhering to established privacy standards, including the US Health Insurance Portability and Accountability Act. Designing more powerful and representative LLMs for clinical use will require increasingly massive clinical datasets, juxtaposing the benefits of large-scale training with privacy considerations. A promising solution is federated learning, which allows LLMs to learn and be aligned from decentralised datasets without direct sharing,^21^ safeguarding data privacy and security. The design and deployment of these LLM-based systems should be guided by the consideration of available computational resources and assessments of the effect of their full lifecycle, including environmental impacts.^9^ Facilitating conversations and existing care presents initial tangible targets for LLMs to improve outcomes now while larger efforts aimed at changes in approach develop complementarily.

### Challenging diagnostic categories

The ability of LLMs to synthesise massive amounts of disparate data presents unique opportunities for advancing prevention and psychiatric nosology. Given that there are no well established biomarkers for any mental illness, and that even the gold standard for diagnostics, the Diagnostic and Statistical Manual of Mental Disorders-5, has variable inter-rater reliability,^22^ the challenge of training LLMs cuts directly into one of psychiatry’s more intractable challenges. Early warning signs and linguistic markers^23^ identified by LLMs indicate their potential for more meaningful clinical stratification and nosology, reflecting the continuum between wellbeing and illnesses.^24^ Despite their potential insights, the sociocultural–technical lens emphasises the need for LLMs to be developed within interpretable clinical-decision support systems. Computerised diagnosis programs have existed for over 50 years and serve as a reminder that the right information alone does not guarantee the right clinical outcome.^20^ Although a new generation of LLM-powered novel diagnostics will not transform care overnight, approaching them as adjunct tools within the broader context of patient care will help ensure that LLMs are effectively integrated into health-care practices.

### Upholding diversity and transparency

Cultural and linguistic characteristics strongly influence expressions related to mental health, posing challenges for LLMs built on English text^8,23^ and western values. Model training often relies on a non-transparent selection of datasets^25^ that potentially contain hidden biases, also making the estimation and comparison of clinical performance challenging. This factor is fundamental because initial findings from preprint papers suggest that LLMs have been shown to offer less comprehensive, less consistent, and less verifiable answers to health-care queries in other languages compared with queries in English,^8^ and provide less empathic support to Black patients.^26^ The use of a transparent list of diverse, multilingual, and representative datasets is a first step towards health equity. Transparency would help to ensure the monitoring of where data are being sourced from, open debate on how to track diversity of data, and inclusion strategies for those less likely to contribute. A second step is to design LLMs that can flexibly align between and within cultural contexts, because symptoms considered pathological in one setting might be seen as normal if not valued in another.^27^ Addressing the challenge of data and value alignment with diverse cohorts will require transparency, public engagement, and the contributions of domain experts, including individuals with lived experiences. Open dialogue with these stakeholders will help to monitor whether LLMs align with societal values and respect the diverse needs of seekers of mental health care.

### Promoting digital inclusivity and literacy

Although the learning health-care system model can help to ensure optimal development and use, the foundation of any technology-enabled system should rest within equitable access. LLMs rely on vast training datasets and biases existing in those datasets will be amplified if attention is not paid to inclusivity at all stages of development and implementation. Although access to the internet is becoming more common, it is still stratified by race, gender, education, and income. Less visible but equally important, digital literacy and skills remain poorly measured and rarely supported in a mental health context. Beyond the fundamental need for LLMs to be trained on diverse datasets to ensure reduced bias, bringing LLMs to diverse communities requires truly embracing the duality of the sociocultural–technical lens. Promoting digital inclusivity across different linguistic and cultural contexts will require directed efforts, including the support of new roles, such as the digital navigator.^28^ These individuals are community members with special training in digital equity, digital health, and digital engagement who will help to ensure all people can use new models of care delivery.

## Conclusion

The integration of LLMs into mental health care presents important challenges, yet the opportunities they offer in enhancing research and care delivery are substantial. We provide model-agnostic guidelines to help design clinical LLMs for the mental health domain. Key recommendations included establishing a global clinical repository for the training and testing of LLMs, establishing ethical frameworks, refining diagnostic constructs, incorporating cultural considerations, and ensuring digital inclusivity. Beyond these key opportunities, the consideration of the sociopolitical context in which the deployment of LLMs will occur is also crucial. Governmental policies will greatly shape how LLMs are accessed across different regions and their ethical governance. For example, accountability should be enshrined in policy, establishing the differential responsibility of developing and deploying organisations in implementing safeguards, addressing adverse outcomes, and evaluating alignment with public health goals. Given their complexity, these topics are beyond the scope of this Viewpoint and require further consideration with support by governmental and health-system stakeholders. Through concerted efforts in addressing these challenges, we can harness LLMs to help clinicians, researchers, and individuals with lived experiences to improve mental health outcomes globally.

## Figures and Tables

**Figure:**
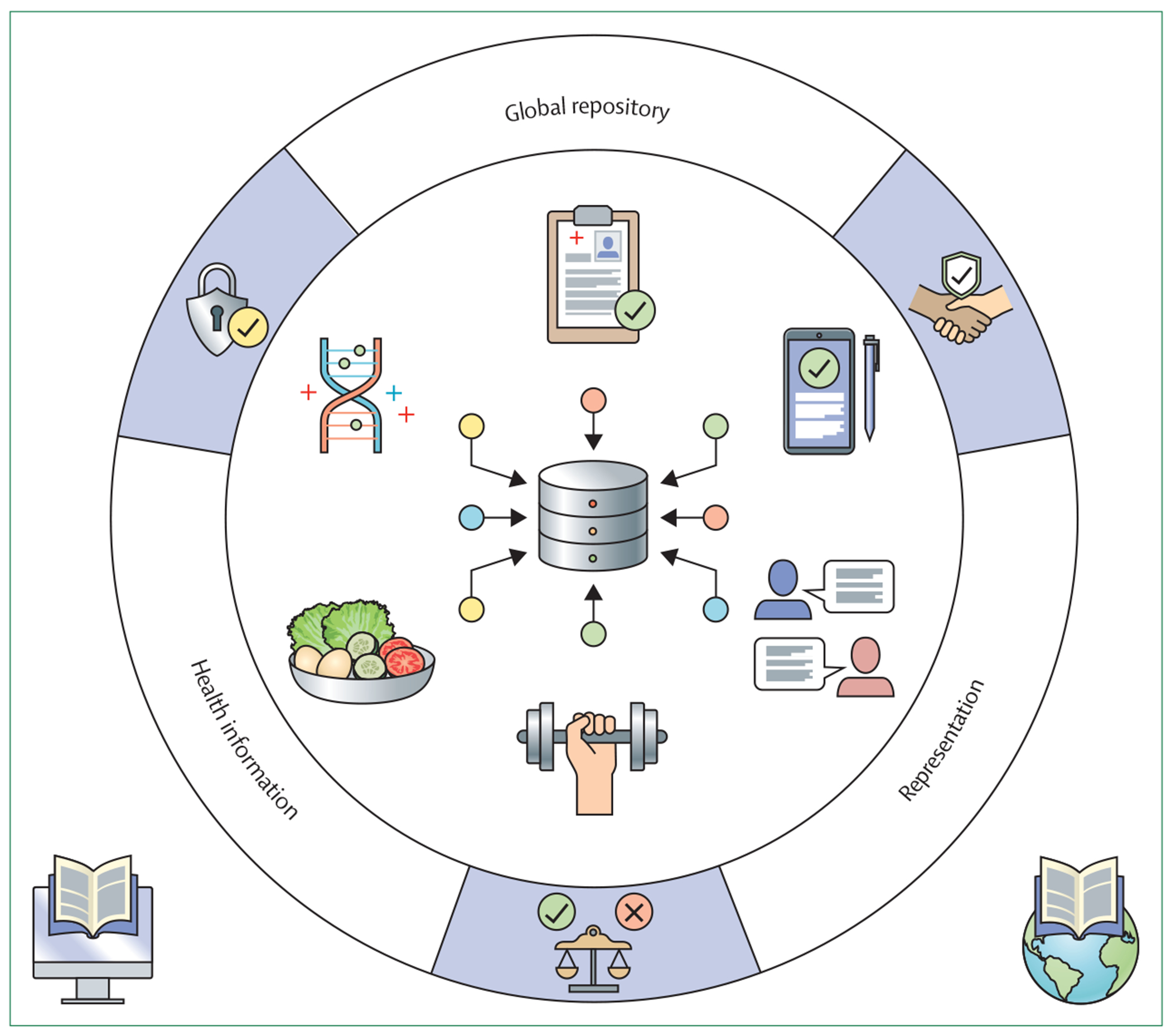
Proposed sociocultural–technical framework A global repository of clinical, biological, and lifestyle data across diverse cultures is used for training and evaluation of large language models. The deployment ecosystem ensures ethical use through data protection, governance, and fairness-building measures. This ecosystem is founded on initiatives for digital literacy and global access to internet and computational resources, with the goal of increasing equitable access.

## Search strategy and selection criteria

We included articles identified through searches of arXiv and PubMed using the search string query: (“large language model”[Title/Abstract] OR “transformer”[Title/Abstract]) AND (“mental health”[Title/Abstract] OR “medicine”[Title/Abstract] OR “psychiatry”[Title/Abstract]). Only papers published from Jan 1, 2017, to July 1, 2024, and written in English were reviewed. Additional relevant articles were included from the convening reading materials and as part of the review process, finalising the reference list based on pertinence to the scope of this Viewpoint.
